# Music performance anxiety: the role of early parenting experiences and cognitive schemas

**DOI:** 10.3389/fpsyg.2023.1185296

**Published:** 2023-06-09

**Authors:** Jennifer Kirsner, Sarah J. Wilson, Margaret S. Osborne

**Affiliations:** ^1^Kirsner Psychology, Melbourne, VIC, Australia; ^2^Melbourne School of Psychological Sciences, The University of Melbourne, Melbourne, VIC, Australia; ^3^Melbourne Conservatorium of Music, The University of Melbourne, Melbourne, VIC, Australia

**Keywords:** music performance anxiety (MPA), Early Maladaptive Schemas (EMS), parenting styles, teaching, treatment

## Abstract

Music Performance Anxiety (MPA) is a common challenge for classical musicians, however its etiology has received minimal research, particularly in regards to caregiver experiences during childhood and adolescence. The aim of this research was to explore the impact of childhood experiences with parents along with patterns of dysfunctional cognitive schemas that develop through childhood ('Early Maladaptive Schemas'; EMSs) on the manifestation and severity of MPA in adulthood. Study 1 employed 100 adult professional, amateur, and tertiary student classical musicians from across Australia. Participants completed the Young Schema Questionnaire (YSQ) and the Kenny Music Performance Anxiety Inventory (K-MPAI). Study 2 included eight participants from Study 1, five of whom scored 1.5 standard deviations or more above the mean K-MPAI score and three of whom scored 1.5 standard deviations or more below the mean K-MPAI score. Participants were interviewed about experiences of parenting during childhood and adolescence, along with their experiences of MPA and musical training. Interpretative phenomenological analysis was used to explore themes in the interview data. Study 1 factor analysis revealed four higher-order EMS factors, *F*_(4, 95)_ = 13.74, *p* < 0.001, one of which was a significant predictor of MPA, *t*_(99)_ = 3.06, *p* = 0.003. This factor comprised themes of failure, catastrophising, and incompetence/dependence. Study 2 qualitative analysis revealed various key parenting themes experienced in childhood that differentiated low- and high-MPA scorers in adulthood. Findings from both studies are discussed in light of clinical applications and interventions, and implications for both parents and music educators.

## 1. Introduction

Research into the phenomenology of music performance anxiety (MPA) has received significant empirical attention, though to-date it has predominantly focused on factors such as symptomatology, demographic and contextual factors, and comorbid psychopathology (Wesner et al., 1990; Cox and Kenardy, 1993; Brotons, 1994; Mor et al., 1995; Huston, 2001; Osborne and Franklin, 2002; Kenny et al., 2004; Smith and Rickard, 2004; Osborne and Kenny, 2005; Iusca and Dafinoiu, 2012; Patston and Osborne, 2016). Despite the dearth of literature exploring the developmental underpinnings of MPA, evidence of the detrimental impact of overcontrolling, overinvolved parenting in young musicians' autonomy, feelings of competence, intrinsic motivation, engagement in music, and general self-efficacy (McPherson et al., 2012; Givertz and Segrin, 2014) suggests that parenting experiences in childhood may have an important role in mitigating or exacerbating the development of MPA. Thus, in this paper we examine the impact of childhood caregiver experiences and the development of maladaptive cognitive frameworks on the emergence of MPA in adult musicians. Study 1 investigated the relationship between Early Maladaptive Schemas (EMSs) and MPA through a quantitative study of adult classical musicians. Study 2 then extended findings from Study 1 through semi-structured interviews of a sub-set of musicians from the Study 1 sample, enabling in-depth investigation into individuals' experiences with primary carers throughout childhood and adolescence.

In order to provide a robust theoretical framework for a developmental investigation of MPA, Barlow's (2000) model of the aetiology and phenomenology of clinical anxiety disorders was adopted (see, Kenny and Osborne, 2006; Kenny, 2011; Osborne, 2015). Barlow outlines three vulnerabilities that can account for development of anxiety or mood disorders: a *generalized biological vulnerability* (or heritability), a *generalized psychological vulnerability* (based on early experiences of developing a sense of control over salient events), and a *more specific psychological vulnerability* (in which an individual learns to focus their anxiety on specific objects or situations). Barlow's model therefore acknowledges the interplay between biological predisposition, early social experiences, and learned response patterns to repeated life experiences that induce anxiety (such as past anxiety-inducing performing experiences). In relation to MPA, Barlow's model provides a foundation to explore the impact of early social learning experiences on the development, experience, and perpetuation of MPA in adult musicians. In the context of MPA research, two prongs of Barlow's model are illustrated by Osborne and Kenny (2008), who found that trait anxiety and gender (*generalized biological vulnerability*) predicted MPA in adolescents, and that this prediction was strengthened by the presence of negative appraisals by young musicians in relation to their perceived “worst” performance experiences (*specific psychological vulnerability*).

This paper outlines two studies that explored the role of early interpersonal experiences with primary caregivers in the development of MPA, and examined how the development of MPA may be shaped by an individual's developing self-concept, beliefs, and perceptions of the social world through their formative years. In order to address the shortfall in research exploring early parenting experiences in the development of MPA, we applied Young et al. (2003) Early Maladaptive Schema model (Young et al., 2003) as a lens through which to explore the *generalized psychological vulnerability* that may precipitate MPA in classically-trained musicians. The research comprised both quantitative and qualitative studies, and investigated the impact of early experiences with primary carers on the development of cognitive schemas (or cognitive organizing frameworks) that are relevant to a musician's perception of their social world and their sense of self. Through this research, we hoped to illuminate core social and emotional processing schemas that might predispose the development of MPA.

Young's Early Maladaptive Schema model (Young et al., 2003) was originally conceptualized to explore the development of interpersonal schemas in early childhood that affect the emergence of complex personality issues and personality disorders in adulthood. The Early Maladaptive Schemas (EMSs) described by Young are closely modeled on Aaron Beck's formulation of cognitive schemas; however, they represent a specific maladaptive or self-defeating subset of the schema concept (Young et al., 2003). For example, whilst healthy, adaptive interpersonal schemas may develop in an individual who has experienced a childhood characterized by secure, healthy attachment and support by primary carers, individuals who have experienced abusive, neglectful, or hypercritical parenting may be more likely to develop maladaptive interpersonal schemas and social expectations that others will mistreat or harm them in some way. As such, Young et al. (2003) defined EMSs as:

Broad pervasive themes or patterns [comprising] memories, emotions, cognitions, and bodily sensations, [relating to] oneself and one's relationship with others, [developing] during childhood or adolescence, [that are] elaborated throughout one's lifetime, and [are] dysfunctional to a significant degree (p. 7).

EMSs therefore serve as templates for the processing of later experiences (Young, 1999). They are triggered by everyday events that are related to the schema's content (e.g., themes around perceived incompetence, shame, fears of criticism or harm), and they are central to an individual's self-concept; particularly self-concept relating to interpersonal context (Young et al., 2003; van Genderen et al., 2012). Individuals may not have awareness of their EMS patterns or the impact of early experiences, and EMSs are likely to be deeply entrenched and automated, particularly in more severe cases. As such, they are difficult to shift, as they become the foundation for self-concepts, personality, and social processing through early social modeling and repeated use (Young, 1999).

EMSs do not always develop as the consequence of early trauma; rather, Young et al. (2003) refers to *dysfunctional* childhood experiences. For example, an individual may develop an EMS relating to perceived incompetence and dependence, which may develop in the context of parental overprotection or over-sheltering during childhood, leading to destructive beliefs around lack of self-efficacy and autonomy as the child matures.

Conversely, Young et al. (2003) describe a range of *core emotional needs* that children require for healthy psychological development. These include: (1) Secure attachments to others (which incorporate safety, stability, nurturance, and acceptance); (2) Autonomy, competence, and a sense of identity; (3) Freedom for the child to express valid needs and emotions; (4) Spontaneity and play; and (5) Realistic limits and self-control. Young et al. (2003) highlight that biological temperament impacts on a child's resilience and response to early caregiver environments; however, according to Young et al. (2003) model, EMSs may develop if core emotional needs are not met in childhood.

Eighteen EMSs have been identified by Young et al. (2003), which may vary depending on the child's temperament and childhood experiences. Young et al. (2003) also outline five theoretically-grounded schema “domains” in which these 18 EMSs are grouped, and each domain corresponds with a specific core emotional need. The 18 EMSs five schema domains, and corresponding core emotional needs are outlined in Table 1.

**Table 1:** Young's early maladaptive schemas.

**Schema domain**	**EMSS**	**Core emotional need**
**Disconnection and rejection**		
Expectation that one's needs for security, safety, stability, nurturance, empathy, sharing of feelings, acceptance, and respect will not be met in a predictable manner. Typical family origin is detached, cold, rejecting, withholding, lonely, explosive, unpredictable, or abusive.	1. Abandonment/instability 2. Mistrust/abuse 3. Emotional deprivation 4. Defectiveness/shame 5. Social isolation/alienation	Secure attachments to others
**Impaired autonomy and performance**		
Expectations about oneself and the environment that interfere with one's perceived ability to separate, survive, function independently, or perform successfully. Typical family origin is enmeshed, undermining of child's confidence, overprotective, or failing to reinforce child for performing competently outside the family.	1. Dependence/incompetence 2. Vulnerability to harm or illness 3. Enmeshment/underdeveloped self 4. Failure to achieve	Autonomy, competence, and a sense of identity
**Impaired limits**		
A deficiency in internal limits, responsibility to others, or long-term goal-orientation. Leads to difficulty respecting the rights of others, cooperating with others, making commitments, or setting and meeting realistic personal goals. Typical family origin is characterized by permissiveness, overindulgence, lack of direction, or a sense of superiority – rather than appropriate confrontation, discipline, and limits in relation to taking responsibility, cooperating in a reciprocal manner, and setting goals. In some cases, child may not have been pushed to tolerate normal levels of discomfort, or may not have been given adequate supervision, direction, or guidance.	1. Entitlement/grandiosity 2. Insufficient self-control/self-discipline	Freedom for the child to express valid emotions
**Other-directedness**		
An excessive focus on the desires, feelings, and responses of others, at the expense of one's own needs, in order to gain love and approval, maintain one's sense of connection, or avoid retaliation. Usually involves suppression and lack of awareness regarding one's own anger and natural inclinations. Typical family origin is based on conditional acceptance: children must suppress important aspects of themselves in order to gain love, attention, and approval. In many such families, the parents' emotional needs and desires – or social acceptance and status – are valued more than the unique needs and feelings of each child.	1. Subjugation 2. Self-Sacrifice 3. Approval-Seeking/Recognition/Seeking	Spontaneity and play
**Overvigilance and inhibition**		
An excessive emphasis on suppressing one's spontaneous feelings, impulses, and choices *or* on meeting rigid, internalized rules and expectations about performance and ethical behavior - often at the expense of happiness, self-expression, relaxation, close relationships, or health. Typical family origin is grim, demanding, and sometimes punitive: performance, duty, perfectionism, following rules, hiding emotions, and avoiding mistakes predominate over pleasure, joy, and relaxation. There is usually an undercurrent of pessimism and worry—that things could fall apart if one fails to be vigilant and careful at all times.	1. Negativity/pessimism 2. Emotional inhibition 3. Unrelenting standards/ hypercriticalness punitiveness	Realistic limits and self-control

Whilst Young et al. (2003) concept of core emotional needs and their role in EMS development has not been empirically validated, there are numerous studies exploring the relationship between early childhood family environments and the role of EMSs as a mediating factor in the development of anxiety and depression. For example, EMSs relating to themes of worthlessness and loss (*Emotional Deprivation, Dependence/Incompetence, Defectiveness/Shame, Failure to Achieve*, and *Social Isolation*) were found to mediate the relationship between childhood adversity and anhedonic symptoms in adolescents (Lumley and Harkness, 2007). The same authors reported that EMSs relating to themes of danger (*Mistrust/Abuse* and *Vulnerability to Harm or Illness*) mediated the relationship between childhood adversity and anxious symptoms. Similar results have been found for the mediating role of various EMSs (*Dependence/Incompetence, Emotional Inhibition, Failure to Achieve*, and *Vulnerability to Harm or Illness*) in the relationship between poor parenting in childhood (low care and high overprotection) and the development of depression in adulthood (Shah and Waller, 2000). Thus, these studies indicate that dysfunctional parenting styles can be important risk factors in the development of adult anxiety and depression, and arguably for the development of adult MPA. Furthermore, as EMSs have been identified as mediating factors, they may play a role in understanding *how* these experiences of parenting styles during childhood may impact on the experience of MPA in adulthood.

The conceptualization of “performance anxiety” can be considered more broadly as a subtype of Social Phobia, referenced within the Diagnostic and Statistical Manual of Mental Disorders (5th ed., text rev.) (DSM-5-TR; APA, 2022) by stipulating a “performance only” specifier subtype of Social Phobia (Social Anxiety Disorder) associated with ‘speaking or performing in public' that includes “fears that are typically most impairing in [the individual's] professional lives (e.g., musicians…)” (Social Anxiety Disorder, Specifiers). This conceptualization is widely supported in literature investigating MPA (Eng et al., 2000; Furmark et al., 2000; Barlow, 2002; Osborne and Franklin, 2002; Osborne and Kenny, 2005; Kenny, 2011). Relationships between Social Phobia more broadly and EMSs have also been identified in prior research (Pinto-Gouveia et al., 2006; Gonzalez Diez et al., 2012), pointing to a similar relationship between EMSs and the presence of MPA, if MPA is understood as a subtype of Social Phobia.

Several studies have also explored relationships between early childhood caregiver experiences and the development of Social Phobia. For example, parental criticism, parental shaming, parental overprotection, and social isolation in childhood, have all been identified as potential contributors to the development of Social Phobia in interaction with the child's temperament (Hudson and Rapee, 2000; Neal and Edelmann, 2003). Similarly, a relationship has been identified between Social Phobia and childhood experiences of parental rejection, overprotection, and lack of emotional warmth (Arrindell et al., 1983). Outpatients with Social Phobia also report significantly elevated experiences of parental overprotection and significantly depleted parental care (i.e., emotional warmth, empathy, and affection) when compared to non-clinical controls (Parker, 1979; Parker et al., 1979; Rapee, 1997; Rapee and Melville, 1997).

These studies indicate that the quality of childhood attachment and early social experiences may be important in the development of Social Phobia, and therefore point to the importance of investigating the role of early experiences in the development of MPA. The identified relationships between EMSs and Social Phobia also suggest that Young's EMS framework may be valuable for conceptualizing the etiology and phenomenology of MPA, and may provide clinicians with a comprehensive yet concise framework for approaching individual factors underlying a client's presenting difficulties (Osborne and Kirsner, 2022).

The aims and hypotheses for this research extended across two studies. In Study 1, we aimed to determine whether a relationship exists between EMSs and MPA, and if so, whether particular patterns of EMSs predict MPA in adults. Findings from Study 1 were subsequently examined in Study 2 through in-depth interviews with a subset of musicians from study 1, with the aim of exploring specific early experiences with primary caregivers, and comparing those with severe MPA in adulthood to those with minimal experiences of MPA.

In Study 1, we hypothesized that a global EMS score (a total score of all 18 EMS scores combined) would demonstrate a significant positive association with MPA scores. Given that previous research has identified a broad range of EMSs associated with relevant constructs such as Social Phobia and anxiety more generally, further exploration of specific groups of EMSs that may serve EMSs as predictors of MPA was exploratory in nature.

In Study 2 we hypothesized that particular aversive experiences of parenting during childhood (such as overcontrolling or neglectful parenting experiences) would be more likely to be reported in adults who experienced high levels of MPA.

## 2. Study 1: the relationship between Early Maladaptive Schemas and MPA

### 2.1. Method

#### 2.1.1. Participants

Participants (*n* = 100) were recruited via social media, email, and advertisements placed within The University of Melbourne, and were required to be 18 years and over, classically-trained, and have five or more years of experience on their instrument. The sample comprised 25 males, 74 females, and one participant who preferred not to provide gender information. The age range of participants varied from 18 to 24 years (13%) through to 65 years or older (1%). A majority of participants were in the 25–34 years (29%) and 35–44 years (30%) age groups, with 17% of participants in the 45–54 years and 10% in the 55–65 years age groups. The sample contained a mixture of full-time (25%) and part-time (24%) professional performers, undergraduate and postgraduate students (14%), amateur musicians (32%), and five participants who had ceased playing due to their MPA (5%). Principal instruments were varied, but were biased toward string players, with 60% of the sample playing a stringed instrument, and the remainder being spread across woodwind (17%), keyboard (8%), brass (4%), percussion (2%), and voice (9%).

#### 2.1.2. Measures

Background information was obtained through a series of questions assessing age, gender, role of music in life (professional, amateur), primary musical instrument, years of experience on instrument, experience of treatment, and use of beta blockers.

MPA was measured using the Kenny Music Performance Anxiety Inventory (*K-MPAI*; Kenny, 2011). This is a 40-item self-report scale that was developed to assess the existence and severity of symptoms associated with MPA on a 7-point Likert scale, ranging from “0 = Strongly disagree”, to “6 = Strongly agree”. Consistent with the measure's theoretical underpinnings in Barlow's tripartite theory of the development of anxiety disorders, principal axis factoring yields three focal areas comprising (1) early relationships and attachment (e.g., “One or both of my parents were overly anxious”, “My parents were mostly responsive to my needs”), (2) general underlying psychological vulnerability (e.g., “From early on in my music studies, I remember being anxious about performing”, “Sometimes I feel anxious for no particular reason”), and (3) performance concern factors (e.g., “Prior to or during a performance, I get feelings akin to panic”, “Thinking about the evaluation I may get interferes with my performance”) (Kenny, 2011). The inventory demonstrates excellent internal reliability in Australian musicians with Cronbach's alpha ranging from 0.86 to 0.95 for one high order and two first-order factors, and equaling 0.94 for the whole scale (Chang-Arana et al., 2017). This indicates appropriate assessment of MPA as a unidimensional construct. Construct validity has been established with Australian professional musicians with highly significant correlations ranging between 0.40 and 0.71 with the State-Trait Anxiety Inventory (Trait), the Social Phobia Inventory, the Anxiety and Depression Detector, and *r* = −0.53 with Core Self-Evaluations (measuring self-esteem and self-efficacy). A cut-off score of 105 on the measure was indicated for clinically significant levels of distress (see Kenny et al., 2014).

EMSs were measured by the Young Schema Questionnaire – Short Form (*YSQ-S3*; Young, 2005), which assesses all 18 EMSs identified by Young et al. (2003). The scale contains 90 items rated on a 6-point Likert scale, ranging from “1 = Completely untrue of me”, to “6 = Describes me perfectly”, with five individual items relating to each individual EMS. Wording was minimally changed for the questionnaire instructions to improve emotional sensitivity for those who may have experienced parental death or parental estrangement (i.e., items that referred to current relationships with parents were adapted to overcome this assumption).

Strong internal consistency (Cronbach's alpha of 0.96 for the overall scale) was demonstrated for the previous 75-item version of the YSQ-S3 (measuring 15 rather than 18 EMSs) in an Australian undergraduate sample (Baranoff et al., 2006). Several non-English translated versions of the current 90-item YSQ-S3 have demonstrated adequate validity and internal consistency for all of the 18 schemas (Saariaho et al., 2009; Hawke and Provencher, 2012; Kriston et al., 2013), with Cronbach's alpha coefficients ranging from 0.66 to 0.94 for individual EMSs across the studies. Psychometric support for the translated versions of the YSQ-S3 mirrors that reported for the English versions of the full-length scale and previous versions of the short-form scale (Steptoe and Fidler, 1987; Welburn et al., 2002).

#### 2.1.3. Procedure

Participants were provided with the link for the online survey on SurveyMonkey, which included a copy of the plain language statement and consent form, the latter of which participants were required to provide by ticking a box indicating their consent. They were first presented with background questions, followed by the K-MPAI and the YSQ-S3. A debriefing statement concluded the online survey. The survey was formatted such that participants were unable to progress through the survey unless all questions were completed. Participants were free to withdraw from the study at any point during their participation. The study was given approval by The University of Melbourne Human Research Ethics Committee.

The statistical procedure was as follows: Means and standard deviations were generated to inspect for outliers and skewness, and Box-Cox transformations (Osborne, 2010) were applied to all variables to account for abnormal distribution of some variables. Correlational analyses were conducted between independent and dependent variables. Exploratory factor analyses were performed to identify broader themes (factors) within the YSQ-S3 items. Multiple regression analyses were then performed to investigate the relationship between each of the YSQ-S3 factors and the overall K-MPAI scores.

### 2.2. Results

Descriptive data are presented in Table 2. Due to skewness in the data for a majority of the EMS scores, Box Cox transformations were carried out on all 18 EMSs to ensure that the data adhered to assumptions of normality for parametric analyses (Osborne, 2010). No significant differences were found in K-MPAI scores for any of the background variables (gender, age, instrument, role of music), and thus all subsequent data analyses were performed on the whole sample.

**Table 2:** Mean, range, standard deviation, and distribution skew z scores for the K-MPAI total score and the YSQ-S3 subscales.

**Measure**	**Min**	**Max**	**Mean**	**SD**	**Skew ratio z score**
K-MPAI	61	186	122.53	27.34	0.5
Emotional Deprivation	5	29	9.85	5.99	6.42
Abandonment/Instability	5	30	10.72	5.76	6.14
Mistrust/Abuse	5	30	11.15	5.77	5.58
Social Isolation/Alienation	5	30	13.23	6.55	2.98
Defectiveness/Shame	5	30	10.26	6.32	5.84
Failure to Achieve	5	29	11.67	6.36	4.68
Dependence/Incompetence	5	23	9.11	4.49	4.77
Vulnerability to Harm or Illness	5	23	10.62	5.08	3.56
Enmeshment/Underdeveloped Self	5	25	8.31	4.39	6.89
Subjugation	5	25	11.41	5.09	3.25
Self-Sacrifice	5	30	17.01	5.17	1.73
Emotional Inhibition	5	29	12.47	5.82	3.07
Unrelenting Standards/Hypercriticalness	8	30	20.1	5.23	−0.92
Entitlement/Grandiosity	5	28	13.33	4.76	3.41
Insufficient Self-Control/Self-Discipline	5	29	12.67	5.45	2.68
Approval-Seeking/Recognition-Seeking	5	27	14.06	5.38	1.91
Pessimism	5	28	12.37	6.05	3.07
Punitiveness	5	30	13.14	5.55	2.9

Standard error of skew = 0.24.

Cronbach's alpha coefficients were calculated for the K-MPAI (alpha = 0.85) and for all YSQ-S3 EMSs (coefficients ranged from 0.71 for *Self-Sacrifice* and *Unrelenting Standards/Hypercriticalness* to 0.93 for *Defectiveness/Shame*). As all alpha coefficients were above the cut-off point of 0.70 (Field, 2009), both scales were considered to have acceptable internal consistency for the current sample and are in line with previous psychometric investigations of each measure (Saariaho et al., 2009; Kriston et al., 2013; Chang-Arana et al., 2017).

Scores for the K-MPAI in the current study ranged from 61 to 186. Interestingly, the mean K-MPAI score (*M* = 122.53, *SD* = 27.34) was significantly higher than in a previously published sample of tertiary flute players (*M* = 68, *SD* = 18.97; *t*_(118)_ = 8.51, *p* < 0.001; Kenny et al., 2011), and professional orchestral musicians (*M* = 83.73, *SD* = 40.72; *t*_(471)_ = 9.00, *p* < 0.001; Kenny et al., 2014).

A global EMS score was calculated by combining the transformed total scores for each of the 18 individual EMSs. As anticipated, this was significantly positively correlated with K-MPAI scores, *r* = 0.57, *p* < 0.001. Correlational analyses also revealed significant positive relationships between the K-MPAI and all 18 EMSs, ranging from *r* = 0.32 to 0.54, *p* < 0.02–0.001 (see Supplementary Table 1). In order to explore latent factors underlying EMS patterns and themes that may predict the development of MPA, an exploratory factor analysis was conducted on the YSQ-S3 subscale scores using the principal axis method with direct oblimin rotation.

Based on eigenvalues and fit statistics, a four-factor model was found to be the *best fit* for the data. All four factors had eigenvalues greater than Kaiser's criterion of 1.0 and in combination explained 69.45% of the variance in MPA scores. The four rotated factors are outlined in Table 3, with 14 iterations required to reach the model. The Kayser-Meyer Olkin measure (KMO = 0.88) verified the sampling adequacy of the data, and Bartlett's Test of Sphericity, χ_(153)_ = 1173.40, *p* < 0.001, indicated that the correlations between items were significantly large to support a principal axis factor analysis. These four factors were labeled according to descriptions of the EMSs provided by Young et al. (2003) and included Factor 1 = *Deprivation/Mistrust*, Factor 2 = *Ego Dysregulation*, Factor 3 = *Inadequacy/Impaired Autonomy*, and Factor 4 = *Undifferentiated Self*.

**Table 3:** Rotated factor loadings of the YSQ-S3 four factor solution.

**Early Maladaptive Schema**	**Factor 1**	**Factor 2**	**Factor 3**	**Factor 4**
Emotional Deprivation	**0.71**	0.41		
Abandonment/ Instability	**0.44**			
Mistrust/ Abuse	**0.54**			
Social Isolation/ Alienation	**0.77**			
Defectiveness/ Shame	**0.71**			
Subjugation	**0.44**		−0.32	
Self-Sacrifice	**0.56**			
Emotional Inhibition	**0.72**			
Unrelenting	**0.58**			
Standards/ Hypercriticalness	**0.46**		−0.32	
Pessimism	**0.60**			
Punitiveness				
Entitlement/ Grandiosity		**0.66**	0.40	
Insufficient Self-Control/		**0.45**		
Self-Discipline		**0.86**		
Approval-Seeking/ Recognition-Seeking				
Failure to Achieve	0.38		**−0.63**	
Dependence/ Incompetence			**−0.70**	
Vulnerability to Harm or Illness	0.38		**−0.33**	
Enmeshment/ Underdeveloped Self				**0.81**

Pattern Matrix data with loadings >0.3 presented. Principal axis factoring with direct oblimin rotation. Factor 1: Defectiveness/Deprivation; Factor 2: Ego Dysregulation; Factor 3: Inadequacy/Impaired Autonomy; Factor 4: Undifferentiated Self.

Tests of normality of the distribution of the four higher order factors were performed to ensure their suitability for further analyses. All factors revealed a skewness statistic of between *z* = 0.02 (Factor 3) and *z* = 1.18 (Factor 2), indicating that they all adhered to assumptions of normality and were appropriate for further parametric testing. Variable numbers were within those recommended by Cohen (1992) for a medium effect size at alpha = 0.05 with adequate power (0.80).

A multiple regression analysis of the four factors on K-MPAI scores, *F*_(4, 95)_ = 13.74, *p* < 0.001, *R*^2^ = 36.70% revealed that *Inadequacy/Impaired Autonomy* was the only significant predictor of MPA, *t*_(99)_ = 3.06, *p* = 0.003 (see Table 4). The individual EMSs loading onto this factor included *Failure to Achieve, Dependence/Incompetence*, and *Vulnerability to Harm or Illness*.

**Table 4:** Multiple regression analysis for MPA and the four EMS factors.

	**B**	**SE B**	**β**
Factor 1	3.55	2.42	0.18
Factor 2	1.5	1.76	0.09
Factor 3	9.65	3.16	0.37^*^
Factor 4	3.68	4.5	0.08

Factor 1: Defectiveness/Deprivation; Factor 2: Ego Dysregulation; Factor 3: Inadequacy/Impaired Autonomy; Factor 4: Undifferentiated Self. R^2^ = 0.37; Adjusted R^2^ = 0.34 (p < 0.01). ^*^p < 0.01.

### 2.3. Discussion

This first study investigated the relationship between EMSs and MPA in order to explore the link between early caregiver experiences and the cognitions and belief systems that contribute to MPA in adulthood. The results revealed significant positive correlations between all 18 EMSs and MPA, and a specific group of EMSs (Factor 3 in the exploratory factor analysis) was identified as a significant predictor of MPA in adult musicians. This group of EMSs included *Failure to Achieve, Dependence/Incompetence*, and *Vulnerability to Harm or Illness*. All three of these EMSs also fall within a broader theme (or “schema domain”) as defined by Young et al. (2003) entitled *Impaired Autonomy and Performance*.

In addition to the EMSs within Factor 3 of the current study, Young et al. (2003) domain of *Impaired Autonomy and Performance* also includes the *Enmeshment/Underdeveloped Self* EMS, which did not emerge within Factor 3 of the current model. Indeed, in the population of musicians investigated in the study, *Enmeshment/Underdeveloped Self* appeared as a stand-alone EMS with its own independent significant factor loading (Factor 4), with minimal loadings onto other factors.

The variability between Young et al. (2003) *Impaired Autonomy and Performance* domain and Factor 3 of the current model it is not unexpected. Young's EMS domains were developed from a theoretical model rather than statistical analyses, and previous factor analytic investigations have revealed mixed support for their robustness (van Vlierberghe et al., 2010; Hawke and Provencher, 2012; Kriston et al., 2013; Bach et al., 2017). Due to the variability between Factor 3 and Young et al. (2003) domain, Factor 3 in the current model was redefined as “*Inadequacy/Impaired Autonomy”*, which we described as ‘*incorporating feelings and expectations of failure in the self, coupled with a sense of uncontrollability/external locus of control and associated feelings of vulnerability'*. This was based on overarching themes of schema content from each of the three EMSs included in the factor provided by Young and colleagues. It appears that for the population of musicians investigated in the current study, issues of individuation and personal identity associated with *Enmeshment/Underdeveloped Self* are independent of the Factor 3 themes and not predictive of MPA.

The inclusion of the *Dependence/Incompetence* EMS in Factor 3 suggests that a lack of practical self-efficacy may be a key component to understanding the EMS structure underpinning MPA. This facet of the current finding is consistent with recent research highlighting the role of self-efficacy in MPA (Egilmez, 2015; Orejudo et al., 2017; Robson and Kenny, 2017; Gill et al., 2022) and anxiety more broadly (Gallagher et al., 2013).

The current study extends previous research by placing self-efficacy in a schematic and developmental context. It suggests that the role of *Dependence/Incompetence* may reflect intrusive and over-involved or over-controlling parenting from a young age, leading to a depletion in a child's developing sense of self-efficacy and autonomy, and a sense of helplessness, dependency, and incompetence as they enter adulthood. This is supported by literature highlighting the role of overinvolved parenting in the development of poor self-efficacy and depleted feelings of autonomy in musicians (McPherson et al., 2012). The impact of these feelings on performance confidence, perceived performance success, and coping skills for MPA may be pronounced, as poor self-efficacy is likely to produce a significant barrier to engaging in effective coping strategies due to beliefs that they are unattainable or futile (Gill et al., 2022).

*Vulnerability to Harm or Illness*, the second EMS in Factor 3, encompasses themes of helplessness and poor self-efficacy similar to that of *Dependence/Incompetence*. However, these themes are often framed in a tendency to worry, catastrophise, and perceive that the world is unsafe and unstable (i.e., they relate to feelings of helplessness due to core beliefs about the world being unsafe or imminent medical or psychiatric emergencies rather than focusing on an individual's perceived incompetence). The combination of *Vulnerability to Harm or Illness* and *Dependence/Incompetence* suggests a particularly debilitating mix of cognitions and beliefs in which an individual may harbor a deep dependency on others due to their poor self-efficacy, coupled with a pervasive belief that others (i.e., the world around them) will not be safe and will not support them. In the context of musical performance, this may manifest as anxiety regarding potential negative repercussions of performance and audience feedback (expectations of harm and catastrophe) coupled with a lack of sense of control or capacity to manage the pressures of the performance adequately (a sense of dependence and incompetence). This has been demonstrated in previous studies in which musicians' “catastrophic” fears were identified as a fear of negative evaluation (Osborne and Franklin, 2002; Osborne and Kenny, 2008), and fear of negative evaluation has been consistently associated as a key component of Social Phobia (Heinrichs and Hofmann, 2001; Stopa and Clark, 2001; Weeks et al., 2005).

The inclusion of *Failure to Achieve* in the current model could be anticipated from previous findings highlighting the strong relationship between perfectionism and MPA (Mor et al., 1995; Patston and Osborne, 2016). Similarly, poor self-efficacy, perceived inadequate preparedness, debilitative perfectionism, and an external locus of control have all been associated with musicians' performance experiences and performance satisfaction (Clark et al., 2014).

The inclusion of *Failure to Achieve* in Factor 3 of the current model further reinforces themes of perceived inadequacy, poor self-efficacy, and an external locus of control. These themes are central to *Failure to Achieve* and similarly underpin *Dependence/Incompetence* and *Vulnerability to Harm or Illness*, and they represent a clear and consistent range of psychological vulnerabilities. These vulnerabilities are likely to develop from a mixture of temperamental factors and early life experiences with primary carers (Young et al., 2003) and together they are predictive of MPA in adulthood. Indeed, various researchers have reported relationships between parental overprotection, hostility, and low levels of parental care in the development of each of the EMSs highlighted in the current model (Shah and Waller, 2000; Harris and Curtin, 2002; Lumley and Harkness, 2007; Haugh et al., 2017).

#### 2.3.1. Early environment predictors of MPA and parenting implications

As previously described, Young outlines several *core emotional needs* that are key to the healthy psychological and social development of a child, and without which a child may be more vulnerable to developing an EMS (Young et al., 2003; Lockwood and Perris, 2012). Given the moderate to strong correlations found between all EMSs and MPA in the current study, Young's aetiological model indicates that parenting experiences early in a child's life warrant further attention in regards to their potential role in the emergence of MPA in adulthood.

According to Young et al. (2003), EMSs from the *Impaired Autonomy/Performance* domain (which includes all Factor 3 EMSs) typically develop from a family background characterized by enmeshment, undermining of a child's confidence, overprotection, or failing to reinforce the child for performing competently outside the family; a proposition that has received empirical support in non-musician populations (Harris and Curtin, 2002; Haugh et al., 2017). Whilst the *Enmeshment/Underdeveloped Self* EMS was not included in Factor 3 of the current model, these general themes of parental overprotection, overcontrol, and undermining of the child's confidence are likely to be similar for individuals who scored high on Factor 3.

Parental criticism, parental overprotection, and parental shaming have also all been previously identified as being significantly associated with the development of Social Phobia (Parker, 1979; Arrindell et al., 1983; Hudson and Rapee, 2000; Neal and Edelmann, 2003; Asbrand et al., 2017) and parental over-involvement has been shown to be significantly associated with the development of anxiety disorders in general (McLeod et al., 2007; van der Bruggen et al., 2008). Given that patterns of overcontrolling parenting have similarly been shown to undermine autonomy, self-efficacy, and intrinsic motivation in young musicians (McPherson et al., 2012), the need to address parenting styles (particularly overcontrol and criticism) to minimize the development and severity of MPA in children and adolescents (and subsequently adults) may therefore be of particular importance.

### 2.4. Study 1 conclusions

By exploring the relationship between EMSs and MPA in adulthood, Study 1 identified typical maladaptive cognitive schema patterns experienced by musicians with MPA. Through this investigation, likely childhood environments associated with those particular clusters of EMSs could be posited based on Young's theory and existing empirical research. Study 1 findings suggest that if core emotional needs of children are not met by primary carers, particularly through overcontrolling or overprotective parenting styles, this may contribute to the development of a combination of EMSs (namely *Failure to Achieve, Dependence/Incompetence*, and *Vulnerability to Harm or Illness*). Collectively these EMSs, which are characterized by expectations of failure, a sense of incompetence and dependence, and an expectation of catastrophe and concurrent sense of vulnerability in adulthood, are a significant predictor of MPA in adulthood.

## 3. Study 2: recollections of childhood experiences with caregivers by adults with MPA

Study 2 extended Study 1 findings by interviewing musicians who experience MPA and explored their early life experiences with caregivers, providing developmental information to contextualize the findings from Study 1.

### 3.1. Method

#### 3.1.1. Participants

Study 2 participants were selected from participants who scored +/- 1.5 standard deviations from the mean K-MPAI score of 122.53 (*SD* = 27.34). This cut-off was chosen to gain an adequate sample size for comparative interviews and meaningful qualitative data analysis, based on willingness and responsiveness of participants from Study 1. The final sample comprised three low scorers (K-MPAI range = 87–91), and five high scorers (K-MPAI range = 148–179), capturing the range of MPA.

#### 3.1.2. Procedure

Participants completed a semi-structured interview comprising open-ended questions that focused on parenting styles highlighted in the EMS research outlined in Study 1 Introduction. Themes included levels and styles of parental involvement, discipline, punishment, love expressed, and encouragement. The interviews ranged from 49 min to 144 min, varying due to the levels of detail, insight, tangentiality, and life experiences of the participants.

#### 3.1.3. Data analysis

Interpretative Phenomenological Analysis (see Pietkiewicz and Smith, 2014) was used to perform a qualitative analysis of transcribed interview data. This enabled a descriptive account of the data provided by participants in relation to their early social experiences, musical development, and current experiences of MPA and EMS patterns. Analyses were performed using qualitative data analysis computer software package, NVivo 11, to assist in drawing out and examining common themes. In presenting the results of the analysis, participant names and non-essential details have been changed to preserve confidentiality.

### 3.2. Results

Participants with high K-MPAI scores demonstrated an overall elevation across EMS scores compared to those with low K-MPAI scores (see Table 5), though variability is evident within individual EMS profiles.

**Table 5:** Demographic data, K-MPAI, and YSQ-S3 scores for Study 2 participants.

	**Low MPA Group**			**High MPA Group**				

	**LowMPA-1**	**LowMPA-2**	**LowMPA-3**	**HighMPA-4**	**HighMPA-5**	**HighMPA-6**	**HighMPA-7**	**HighMPA-8**
Gender Age (years) Instrument type Experience (years) Role of music	F 35-44 Strings 21+ Professional full-time	F 25-34 Woodwind 21+ Amateur	M 35-44 Woodwind 21+ Professional part-time	F 25-34 Strings 21+ Professional full-time	F 35-44 Strings 21+ No longer playing	F 45-54 Strings 21+ Amateur	F 55-64 Woodwind 21+ Professional part-time	M 55-64 Keyboard 21+ Amateur
MPA^*^	90	87	91	179	150	148	161	155
Emotional deprivation			2.6		2.6	2.8	2.6	5.6
Abandonment				3.6				2.6
Mistrust/abuse					3.4		4.4	
Social isolation					3.6	4.6	5.0	4.6
Defectiveness/shame							3.8	3.4
Failure to achieve		2.2		2.8	5.6	3.2		2.6
Dependence/incompetence					3.4			
Vulnerability to harm				4.0	3.6		2.8	2.8
Enmeshment								
Subjugation					4.6	3.0	3.6	3.4
Self-Sacrifice	3.2	3.0	3.2	3.4	4.8	4.2	3.2	3.0
Emotional inhibition						3.8		
Unrelenting standards	3.2	4.4	4.0	4.8	3.8	3.6	3.6	4.4
Entitlement		3.6		2.8		2.8	5.4	
Insufficient self-control				4.6	4.0			3.8
Approval-seeking		5.0		3.2	2.8		4.8	3.2
Pessimism				3.8	3.6	2.8	3.0	3.6
Punitiveness				3.8	3.2		4.2	3.8

Only the YSQ-S3 scores that exceed Axis I clinical outpatient norms provided by Hawke and Provencher (2012) are provided.

^*^MPA (K-MPAI) mean score for overall Study 1 sample = 122.53 (SD = 27.34).

#### 3.2.1. Key parenting themes

Key parenting themes were explored in the data (see Table 6), and comprised various aspects of parenting styles and behaviors, including levels and types of parental involvement, regulation and stability of parental emotional expression, and the way in which love is expressed. Table 6 outlines these key parenting themes alongside examples of associated adaptive and maladaptive home environments as described by participants.

**Table 6:** Study 2 core themes and adaptive and maladaptive descriptions identified in the qualitative analysis.

**Key parenting theme**	**Adaptive home environment**	**Maladaptive home environment**
Affection/expression of love	Overt demonstrations of affection and love (physically, verbally, or other non-verbal demonstrations) apparent to child	Lack of affection or conflicting messages of love, child feels unloved
Soothing and safety	Provision of soothing when a child feels afraid or distressed, provision of a ‘safe, stable base'	Emotional needs not met, child feels unprotected and isolated
Parental involvement	Parents' active and non-judgemental involvement in the child's daily life and musical activities	Child's practical daily needs and interests not supported
Parental encouragement and support	Demonstrations of emotional support, unconditional praise and encouragement	No support or encouragement, parent is disinterested or critical, or encouragement is provided conditionally
Parental expectations	Parents provide unconditional support of child's intrinsic motivation and interests, support of the child's engagement and attempts	Comparisons of child to peers, parental investment in musical outcomes or own agendas, focus is only on child's successes
Parental anger and rage	Predictable and stable mood in parents, appropriateness of parental emotional responses to disciplining and their own stress	Demonstrations of angry behavior (e.g., explosive verbal outbursts), criticism, unpredictability of outbursts

##### 3.2.1.1. Affection/expression of love

This key parenting theme relates to a child's consistent knowledge that they are loved and valued, whether this is expressed by caregivers physically, verbally, or via other more indirect means.

Participants with high MPA described home environments typically characterized by varying degrees of emotional or practical neglect. Demonstrations of love or affection were often provided unreliably or were coupled with other needs not being met:

Of course, we were all loved, but we were never told it. No one was ever told “I love you”, ever…I can remember when I was a little kid, [Mum] would always be standing at the sink washing the dishes, and coming up behind her and just holding her around the legs or around the waist or something like that, giving her a hug like that, and she never really accepted it easily…So long as you weren't being yelled at, the rest of the time you were meant to infer that things were ok and everything was alright. [HighMPA-7]

Another respondent described contradictory and varied memories of his mother during his childhood, responding at one point:

I don't remember any physical contact with my mother. [HighMPA-8]

At another point, the respondent described his mother's soothing and physical affection when he was distressed following a soiling incident as a young child, stating:

Mum was very affectionate, and she still is. [HighMPA-8]

However, this participant also described violent, unpredictable episodes of rage by his mother at other times (see *Parental Anger and Rage*), reflecting strongly conflicting and unstable messages of love and affection provided by her.

In contrast, participants with low MPA described reliable and stable experiences of affection and love in their home environments, whether expressed physically, verbally, or demonstrated less overtly:

[My parents] were very affectionate people…We always kiss each other. We're always saying “I love you” …I'm very blessed in that I had a really supportive and loving family. [LowMPA-2]

[My parents have] always been very loving and very affectionate. My Dad was always demonstrably affectionate with his two sons...Mum's a very loving person. A huge capacity for love. So I would describe my upbringing as loving. That's the word...We were definitely told explicitly that we were loved. And shown physically. [LowMPA-3]

[My parents were] definitely supportive and affectionate. They weren't very physically or verbally affectionate, but they were very supportive. But neither of them are particularly demonstrative people…I felt safe and very looked after. [LowMPA-1]

##### 3.2.1.2. Soothing and safety

This key parenting theme relates to the availability and consistency of soothing provided by parents to enable a child to form a safe and stable emotional base from which they can develop confidence and experience the world as a safe and supportive place.

Many high MPA participants described experiences in which their parents were not consistently available for emotional support or soothing. The impacts of these reported experiences are similar to those of lack of parental affection, as they appear to have contributed to participants' developing feelings of anxiety, lack of self-efficacy, poor self-worth, self-reliance, and sense of uncontrollability or unreliability about the world around them.

I'd never go to my Mum [for affection]. I went to her once about something and the [shit] really hit the fan. She couldn't cope…I don't remember any physical contact with my mother…My brother and I were always on edge. [HighMPA-8]

My bedroom was right at the end [of the corridor] and I was afraid of the dark. Mum would say “You have to go to bed now” and I'd say “I can't go out there; It's all dark” and then she'd go [to my sister] “Oh God, take [your sister] out there. She's scared of the dark!” She used to make out like I was a pain in the neck. [HighMPA-7]

So I was 14 months old when [my next younger sibling] was born and I think she was quite a needier kid than I was as a baby, so I think I probably just learned to be quite self-contained because she cried a lot and needed attention as a baby and I was just happy to play by myself or whatever.…And my youngest sister was premature so she got a lot of attention obviously as a little one…A 14-month-old kid is a baby. You don't want to be left to do your own thing at 14 months. The baby doesn't want to be left alone. But that was out of necessity. How do I think that affected me? I think I've just learned to become a bit more self-contained. Not expecting that someone's going to come and entertain me, or attend to me straight away. I tend to as an adult, as a result of that, I look for my own solutions really…I used to get scared a lot in the night as a kid. I thought there were boogeymen out the window and sharks under the bed…if I woke up and felt scared, I would jump out of the bed wide enough to not get caught by the wolves or sharks under the bed and then I'd go and just sit next to Mum and Dad's bed on Mum's side, just to be close to them. But I wouldn't always wake them up…because I didn't want to intrude really. So I'd just sit there and wait and sometimes, after a while, Mum would wake up and see me sitting there. [HighMPA-6]

Of the low MPA participants, memories of soothing and safety were expressed in differing ways, but each participant described a safe, stable base.

[My Mum] was a nurse, so she had that kind of capable thing as well. She was very practical and capable. If I was in tears, [I'd go to] my Mum. Definitely my Mum. Physical comfort. Definitely affection. So I felt safe and very looked after. [LowMPA-1]

[I would go to] my Mum. Hugs and kisses and pats on the back and being told it's all ok. [LowMPA-3]

##### 3.2.1.3. Parental involvement

This theme relates to parents' active and non-judgemental involvement in the child's daily life. It incorporates involvement of parents in day-to-day practical parenting tasks (such as meals, bedtime stories, after school care) along with involvement in the child's learning, both in school and extracurricular activities.

Several participants with high MPA recalled their parents not providing for them or supporting them adequately in practical day-to-day needs:

At home, when we were playing, our Mum never played with us at all. Didn't use to read us stories at night. [HighMPA-6]

I just don't remember that [my parents] were around that much…I remember Dad doing a lot of the cooking, but it was very basic ‘soup a la Dad': Brown some mince, put a couple of cans of soup in and a chunk of frozen mixed vegetables. Ready in 20 minutes, and then they'd both be off…They would have been able to put us to bed I guess. We would come home from school and there would be babysitters…Mostly we'd just sit there and watch telly or whatever. [HighMPA-5]

This was coupled with recollections by some high MPA participants that they were not taught basic daily living skills that would have assisted with increased autonomy and confidence as they entered adulthood:

[My mother] just did stuff. She certainly didn't delegate. Even when we got older, she just did it all. I never learned how to cook until I moved out of home. Even the washing. We would say “Mum, why don't you get us to put on the washing?” and she just never did…A bit of a martyr syndrome thing going on…I felt angry because it did impact on us…because in a way, she projected her feeling of martyrdom and we had to carry the burden of being the burden for her. And I felt angry about that. [HighMPA-6]

We had a cleaning lady who would come, but I didn't really learn how to…I had no idea when I left home how to operate a washing machine…I was pretty useless as a human being other than I could play the piano reasonably well. [HighMPA-5]

Conversely, the strong focus on music practice was a theme in the high MPA participants:

[Mum] tells me now that ‘I was expecting you to practice every day, so I felt like that was enough'. That she didn't expect us to tidy our rooms as well. Personally, I think that's a mistake. I think she's got it topsy turvy. [HighMPA-5]

Parents of high MPA participants were in some cases remembered as having critical and/or personal involvement in their child's music practice and achievements, or in other cases being completely disengaged and disinterested:

I know at one point [my mother] had 100 students [as an instrumental music teacher]. On top of that was this eisteddfod and running her violin ensembles…maybe she just couldn't say no. If someone wanted to learn violin, she felt obliged to teach them because she felt if she didn't do it, there was no-one else. But her loyalty was there…One thing I have wondered is, on some level I was made to feel I was somehow special by my parents, by my Mum. I think by not having to do chores around the house or contribute to the household but also being always made to practice…because of this musical training…I felt like I was – it sounds really embarrassing and juvenile – that I was special in some way. I had this underneath idea that I was better than other people because I played an instrument…But at the same time, I had this feeling of total incompetence or worthlessness. [HighMPA-5]

[Mum] never came to any lessons, ever [and] Dad had very little part in my life. He was like a background shadow…[but] the classic thing was you'd be doing your piano practice and [Mum would] be in the kitchen doing something like the dishes or making the dinner or whatever, and then she'd just go insane! She'd go “No! No! That's wrong! That's wrong!!” and she'd come running into the room and say “That's wrong! You have to do this!” And you'd have to do that! [HighMPA-7]

In contrast, all the participants with low MPA recalled involvement by their parents in daily activities and daily life, describing encouraging and safe environments, actively supporting intrinsic motivation and valuing family connection:

My poor father was a taxi driver [driving me to rehearsals and concerts throughout the week]… We [also] played sport together as a family every week. From my early teens until I left home, we played badminton and table tennis every Saturday night at the local sports center. [LowMPA-2]

##### 3.2.1.4. Parental encouragement and support

This key parenting theme relates to the demonstrations of non-judgemental encouragement, emotional support, and unconditional praise provided by parents. Participants with high MPA frequently described only conditional praise when reflecting on their parents' provision of encouragement and support:

It seemed like you'd only get attention if you did something good…You'd get told off enough, but you wouldn't get positive affirmations. It felt like the only time that you'd get positive affirmations was when you do something that my Mum could brag about. I think both my Mum and Dad were insecure and felt inferior, so the fact that she'd have something so she could say “My daughter's better than you”. [HighMPA-7]

Other high MPA participants reported of a lack of encouragement expressed by parents entirely:

[During music practice] I remember some pretty awful times actually. I remember us both screaming at each other. [Mum] crying but still sitting next to me, making me do it. I don't remember any nice things about that. [HighMPA-5]

Of the low MPA participants, experiences were described as consistently supportive, free from specific expectations by parents, and encouraging of freedom and autonomy:

They expected us to be quite independent. But they were very supportive of things that we wanted to do. I never felt like it was their expectation for doing things that was driving any of the things that we did at school. There was very much a feeling of what we did was up to us. [LowMPA-1]

My parents] always encouraged me and my brother as well, to do whatever we liked, and whatever interested us to follow up to the nth degree. Very supportive. Although they weren't from a musical family themselves. [LowMPA-3]

##### 3.2.1.5. Parental expectations

This key parenting theme relates to parenting styles that focus on particular vested interests and outcomes rather than a child's intrinsic motivation. It was often implemented through overcontrol and criticism, and frequently incorporated comparisons with other children.

Of the high MPA participants, expectations regarding music training were high, though they were associated with differing parental agendas:

Mum was the president of the [local] eisteddfod, so she ran this big music competition every year…and so I was made to go in it every single year. Most of the time I wouldn't even get a place. And I just remember, I would just be devastated and howling on the bed, and it was just awful…I think there was this pretty strong message there with how I performed was linked to my value as a person, to be honest. Because not very much time was spent with my [parents] other than being concerned with whether or not I had practiced. [HighMPA-5]

I think my Mum had this idea that we had to be ‘perfect ladies'. She used to say that to me and my sisters and had this idea of what perfect ladies should be. We had to have gloves, fancy dresses, a handbag. I never really knew what the handbag was about. I'd say to her “What's a handbag for?” and she'd say “You have to keep your hanky and your bottle of perfume”. This was in primary school, from probably around eight or nine…Part of ‘being a lady' was that you had to be able to play the piano.” [HighMPA-7]

[I remember one time] where I couldn't find my school shoes and I hadn't practiced so I wasn't allowed to go to school, and by the time I'd got to school, I remember having to be taken out of the classroom very quickly because I must have been hysterical. I was about seven. And I remember telling the teacher that Mum had told me that if I didn't do my practice and didn't do things properly I wouldn't get to go to university. [HighMPA-4]

In contrast, all participants with low MPA uniformly recalled their parents' unconditional support of their interests and autonomy:

It never felt like I should have done better in [Mum's] eyes. She was pretty unconditional about that. If this is what you want to do, then we'll support it. I never felt like I wasn't good enough…I never felt like I was letting my mother down with anything that I did. As long as I tried, I think she was really fine with that. [LowMPA-1]

My parents didn't push me to do stuff…I really loved school and the learning experience, and the group dynamics of playing in ensembles and the solo instrument as well. That appealed to me as well and I got good at it quite quick. [LowMPA-2]

##### 3.2.1.6. Parental anger and rage

This key parenting style relates to experiences of unpredictable and/or explosive anger outbursts by parents, which were described by several participants with high MPA:

We were really scared of our Mum. My parents had their own mental health problems, so I can remember coming home and my older sisters would say “Mum's in a bad mood!” and we'd go somewhere and hide because we didn't want to get near her. Her rage was just really, really scary. Yeah, she would hit us. But was mostly her rage that you were most afraid of…Basically I've grown up feeling that I'm a bad person and I find it really, really hard to throw that off. That I'm always worrying that I'm going to be criticized and what I've done wrong now…I think I just go about with the expectation that people aren't going to accept me for what I am. [HighMPA-7]

My Mum is quite a highly anxious person. Very, very stressed, and has depression. I think that was all undiagnosed when we were small and that's come to light. Lots of conversations about that stuff now and how it has affected us. Very, very angry. Lots of anger…I think that was her way of expressing things. Her struggles came out in quite an angry way and often directed at us. I remember being in trouble a lot. I would get smacked a lot. I was quite scared of her because of that side of her personality. I remember being very stressed and very upset as a child. I remember a lot of crying and a lot of sick in your stomach feeling because of the tension in our house. There was a lot of fighting…I think as a child I just would have felt a lot of the time that I was bad and naughty, and I don't know if that's actually true, but I do remember feeling guilty that I was bad as a child. I think I have the capacity to take things on and feel responsible for them. I feel guilty and like ‘Could I have done that better? Is it my fault?'. [HighMPA-4]

Mum is terrifying and wonderful at the same time…So, things were sometimes quite frightening when I was a kid…Mum was violent. She suffered quite badly from rages [HighMPA-8]

When [Mum] was angry, it was never outwardly expressed. She was a passive aggressive angry person. She'd slam cupboard doors and bang pot lids and you'd stay out of her way until she went and got more normal again. We didn't deal with emotional stuff outwardly at all when I was growing up. [HighMPA-6]

### 3.3. Study 2 conclusions

Broadly, the developmental etiology suggested by the current findings is consistent with prior research highlighting the role of overcontrolling, neglectful, and critical parenting in the development of EMSs (Shah and Waller, 2000; McGinn et al., 2005; Haugh et al., 2017). Findings also reflect previously established associations between parental criticism, parental overprotection, and parental shaming and the development of Social Phobia (Parker, 1979; Arrindell et al., 1983; Hudson and Rapee, 2000; Neal and Edelmann, 2003; Asbrand et al., 2017). Furthermore, the impact of parental expectations and overcontrol has been noted in parents of young tennis players, for whom the pressures of elite performance bear many similarities to those of performing musicians (Gould et al., 2006).

Findings similarly reinforced prior research identifying the detrimental impact of parental overprotection and overcontrol on the development of autonomy and intrinsic motivation in children (Ryan and Deci, 2002; McPherson et al., 2012; Haugh et al., 2017). In the current study, parental expectations relating to children's music goals and parental aggression and control associated with music practice described were frequently described as contributing to poor identity development outside music, poor autonomy, poor self-efficacy, high anxiety, and feelings of inadequacy by participants with severe MPA.

The conditional praise and frequent criticism described by high MPA participants similarly appeared to impede development of confidence, self-efficacy, intrinsic motivation, and a robust and identity outside of music. The impact of parental support (practical and emotional support, and support of developing autonomy) on the ongoing engagement and enthusiasm for music training in children is well established (McPherson and Davidson, 2002), and the current findings begin to shed light on the longer-term impacts of overcontrolling, neglectful, and critical parenting on musicians as they enter adulthood, both in terms of the development of MPA and broader mental health difficulties. The recollections of parental overcontrol coupled with poor parental support echo the description of Barlow (2000) “psychological vulnerability” in the context of MPA development as described by Kenny and Osborne (2006), who suggested that MPA may develop in part as a result of a home environment in which parental expectations for excellence are high but support for achieving excellence is low. Participants high in MPA who described parental aggression and verbal abuse in childhood also reflected on the role of these experiences in producing a constant sense of fear and anxiety, distress, and poor self-worth. A wealth of prior research has identified that parental verbal abuse, aggression and antipathy (including rejection, criticism, and hostility) predict anxiety, depression, self-criticism, fears of rejection, and separation anxiety in adolescents and adults (Sachs-Ericsson et al., 2006; Teicher et al., 2006; Schimmenti and Bifulco, 2015).

In consideration of the key parenting styles identified in Study 2, the first three of Young et al. (2003) *core emotional needs* appear to be particularly applicable when exploring aetiological vulnerabilities associated with the development of MPA, namely, (1) Secure attachments to others; (2) Autonomy, competence, and a sense of identity; and (3) Freedom for the child to express valid needs and emotions. These *core emotional needs* align closely with the content of the five key parenting themes, and further support the impact of a *generalized psychological vulnerability* as proposed by Barlow (2000). Through the lens of Barlow's model, MPA may in part stem from childhood experiences in which the opportunity for the development of a sense of control over salient events is absent. These experiences may be characterized by a lack of stability and nurturance, emotional neglect, unpredictable, explosive anger, and a lack of parental support for the development of autonomy and identity (through extrinsic motivation, poor support for a child's intrinsic interest and needs, and parental overcontrol). As proposed by Barlow and supported by the high MPA participants in Study 2, these early environments appear to contribute not only to the development of broader mental health difficulties and dysfunctional cognitive styles (as demonstrated by elevated EMS score profiles) but most pertinently to the development of debilitating MPA.

Study 2 clarifies what children and adolescents do need for healthy psychological development and minimizing the development of MPA; namely, the provision of parenting styles that were absent in the participants who experienced severe MPA and present in those who experienced minimal MPA. These include active parental involvement and availability in the child's life and musical training, but this must be in a facilitating environment that engenders the development of intrinsic motivation and autonomy in the child, and provides them with an opportunity to develop their own sense of identity; in the words of McPherson (2009), to provide “scaffolding” for the child's own developing interests. Additionally, an emotionally secure, stable home environment in which the parent is consistent in providing appropriate encouragement and affect in their responses, is important for the child to develop feelings of safety and control in the world, and to develop their sense of self-worth and self-efficacy.

## 4. Discussion

These studies investigated a potential aetiological pathway from parenting experiences in childhood and adolescence to the development of cognitive and social schemas relevant to MPA in adulthood. Using a quantitative analysis, Study 1 provided support for the role of EMSs in the development and experience of MPA, particularly those associated with themes of failure, expectations of harm or catastrophe, feelings of vulnerability, and a sense of incompetence and dependence. Through an in-depth qualitative interview, Study 2 extended these findings by identifying key parenting themes that appear to be associated with vulnerability to the development of MPA in adulthood. These key parenting themes include Affection/Expression of Love, Soothing and Safety, Parental Involvement, Parental Encouragement and Support, Parental Expectations, and Parental Anger and Rage. Whilst the findings from both studies cannot provide a causal relationship between parenting styles, the development of EMSs, and the development of MPA, they provide a strong foundation for particular themes and vulnerabilities that may typically be associated with such an aetiological pathway.

### 4.1. Implications

By increasing our understanding of the effect of early social experiences on the development of MPA, there are implications not only for clinical intervention, but for shaping early home environments and music education environments to support young musicians in building resilience and minimizing the development and impact of MPA.

#### 4.1.1. Parenting implications

While Study 1 revealed particular EMSs that were collectively predictive of MPA, variable EMS profiles were evident in the small sample of high MPA participants in Study 2. Nevertheless, at least one of the five key parenting themes was evident in each of the high MPA participants, and each were markedly different in their accounts of childhood parenting experiences from those in the low-MPA group.

Practical support and interventions for parents may take the form of education by music teachers and clinicians regarding the parents' need for an active, non-judgemental role in a child's music practice, their support and encouragement of a child's intrinsic motivation and engagement in music, and the importance of fostering autonomy and confidence. Given the associations between critical and overprotective parenting styles and anxiety disorders more generally, the need for parents to adopt healthy and adaptive parenting styles beyond a child's musical education may be vital not only in mitigating the development or severity of MPA in adulthood, but also in supporting the child's developing resilience and psychological wellbeing.

#### 4.1.2. Instrumental learning implications

The impact of teachers may be understood as secondary to that of parents as their role in the child's life is less involved and more focused. Nevertheless, children and adolescents who learn a musical instrument are likely to spend significant time with teachers and in music education programs if they are thoroughly engaged in their learning and if they are likely to continue on their musical trajectory either as an amateur or professional musician (McPherson et al., 2012). As such, the relationships with key individuals and the social environments experienced whilst learning music are likely to be important factors in developing resilient musicians who are able to manage the pressures of performing and remain connected to the intrinsic joys of music-making. As with parenting practices, the current study highlights the need to foster autonomy, confidence, and self-efficacy within these environments whilst providing non-judgemental support and encouragement, all of which have been previously identified as important in improving music students' motivation and engagement (McPherson et al., 2012). Teachers may also have the capacity to mitigate the impact of damaging parenting influences such as criticism, lack of interest, or overcontrol through psychoeducation and modeling as described above.

#### 4.1.3. Treatment implications

Findings suggest that identification of individual EMS profiles may be of particular assistance in clinical settings when addressing the aetiological underpinnings of MPA presentations in adults. While Study 1 revealed overall patterns of EMSs that predicted MPA, the diversity in individual EMS profiles observed in the small Study 2 sample is consistent with prior research demonstrating the breadth of EMSs that can be associated with particular parenting experiences (e.g., Harris and Curtin, 2002; Lumley and Harkness, 2007; Haugh et al., 2017). Nonetheless, both studies indicate the value in investigating early life experiences and EMS profiles when treating MPA in adults. In this clinical context, Young's Schema Therapy approach (Young et al., 2003) may provide a valuable therapeutic framework to enable dysfunctional interpersonal patterns and belief systems about the self to be reshaped and healed through.

Schema Therapy incorporates the therapeutic relationship as a model for healthy interpersonal functioning and addressing core emotional needs, assisting the client in identifying past dysfunctional schema patterns, and developing adaptive coping styles to recover from past dysfunctional schema patterns (Young et al., 2003). Schema Therapy has gained empirical support as an effective treatment for personality disorders (Sempértegui et al., 2013; Bamelis et al., 2014; Arntz et al., 2022) and cautious support as a treatment for Axis I disorders (van Vreeswijk et al., 2014; Peeters et al., 2021; Straarup et al., 2022). As such, Schema Therapy warrants consideration as a potentially effective psychodynamic therapeutic approach for MPA, but further empirical support is needed. Whether or not a semi-structured treatment such as Schema Therapy is adopted, however, the understanding of a client's underlying EMS profile may still illuminate underlying treatment needs for a client with elevated MPA levels. For example, Study 1 indicates that themes of helplessness, a lack of self-efficacy, and beliefs and expectations of failure are significantly predictive of MPA in adult amateur, student, and professional musicians. As such, understanding and working with confidence, autonomy, self-efficacy, and personal strengths may be of particular assistance in reducing long-term vulnerabilities to MPA over-and-above practical strategies to assist with performance-based skills and relaxation.

### 4.2. Limitations and further explorations

Whilst robust quantitative and qualitative findings emerged from the current studies, there are several limitations identified that may assist in further consolidating and extending findings.

Firstly, Study 1 had an adequate sample size for statistical power (Cohen, 1992). However, replication with a broader and larger sample would be of benefit in providing further support for the findings, and would enable an assessment of the role of individual EMSs in the development of MPA rather than providing only a predictive model of higher-order factors.

Secondly, additional psychometric data illustrating retrospective accounts of early parenting experiences would enable analysis of a broader adult sample for future research. For example, replication of Study 1 with the inclusion of the Young Parenting Inventory (YPI; Young, 2014); a psychometric measure that specifically assesses retrospective accounts of parenting experiences in childhood that are thought to be associated with particular EMSs, would deepen our understanding of the relationship between childhood parenting experiences, EMSs, and MPA in adulthood.

Thirdly, an examination of EMS constructs in more depth would enrich the current findings; in particular, an investigation of the potential role of schema “modes” (referring to the active manifestation of an EMS and its associated coping response; Young et al., 2003) during pre-performance and performance experiences, and primary and secondary pathways to EMSs.

Finally, further studies may benefit from including an assessment of psychiatric disorders (particularly Social Phobia) which may assist in elucidating/differentiating more generalized risk factors from early childhood parenting experiences and those that are more specifically associated with the development of MPA.

## Data availability statement

The datasets presented in this article are not readily available because participants of this study were not asked to consent for their data to be shared publicly. Requests to access the datasets should be directed to mosborne@unimelb.edu.au.

## Ethics statement

The studies involving human participants were reviewed and approved by the University of Melbourne Human Ethics Research Committee. The patients/participants provided their written informed consent to participate in this study.

## Author contributions

JK designed and executed the study, performed the analysis, and wrote the manuscript. SW and MO collaborated in the study design, methodology, analysis, and writing of the paper. All authors contributed to the article and approved the submitted version.
