# Supplementary material for: Music performance anxiety: the role of early parenting experiences and cognitive schemas
Source: Front Psychol. 2023 Jun 9;14:1185296. doi: 10.3389/fpsyg.2023.1185296 (PMC10288882; doi:10.3389/fpsyg.2023.1185296)
Supplement: Supplementary file 1 [file Data_Sheet_1.PDF]

Supplementary Material

Music performance anxiety: the role of early parenting experiences and cognitive schemas

Jennifer Kirsner, Sarah J Wilson, Margaret S Osborne*

*** Correspondence:** Margaret Osborne mosborne@unimelb.edu.au

Supplementary Table 1 Correlation Matrix of the K-MPAI and Transformed YSQ-S3 Variables

	ED	AB	MA	SI	DS	FA	DI	VU	EN	SU	SS	EI	US	ET	ISC	RS	PE	PU
MPA	.32**	.46**	.42**	.45**	.43**	.47**	.45**	.54**	.33**	.32**	.33**	.37**	.36**	.24*	.44**	.32**	.54**	.35**
ED		.45**	.46**	.49**	.58**	.32**	.27**	.34**	.13	.44**	.37**	.47**	.38**	.41**	.33**	.24*	.44**	.42**
AB			.57**	.63**	.57**	.48**	.51**	.58**	.25*	.56**	.28**	.42**	.38**	.49**	.36**	.57**	.70**	.52**
MA				.73**	.57**	.48**	.40**	.63**	.38**	.54**	.50**	.45**	.38**	.47**	.48**	.42**	.66**	.50**
SI					.73**	.48**	.45**	.58**	.36**	.64**	.46**	.63**	.51**	.42**	.36**	.29**	.69**	.51**
DS						.56**	.56**	.53**	.26**	.57**	.36**	.64**	.46**	.30**	.48**	.35**	.66**	.61**
FA							.68**	.53**	.19	.55**	.41**	.44**	.35**	.05	.45**	.23*	.58**	.56**
DI								.62**	.38**	.64**	.19	.36**	.37**	.22*	.49**	.48**	.60**	.48**
VU									.46**	.58**	.32**	.47**	.37**	.39**	.54**	.44**	.80**	.54**
EN										.44**	.36**	.34**	.23*	.32**	.35**	.31**	.37**	.22*
SU											.36**	.60**	.42**	.26**	.37**	.36**	.58**	.53**
SS												.40**	.36**	.24*	.25*	.06	.35**	.40**
EI													.50**	.28**	.34**	.19	.54**	.52**
US														.33**	.38**	.25*	.43**	.65**
ET															.46**	.54**	.38**	.27**
ISC																.58**	.49**	.48**
RS																	.50**	.35**
PE																		.66**

Note. * $p < .01$, ** $p < .001$. ED = *Emotional Deprivation*, AB = *Abandonment/Instability*, MA = *Mistrust/Abuse*, SI = *Social Isolation/Alienation*, DS = *Defectiveness/Shame*, FA = *Failure to Achieve*, DI = *Dependence/Incompetence*, VU = *Vulnerability to Harm or Illness*, EN = *Enmeshment/Underdeveloped Self*, SU = *Subjugation*, SS = *Self-Sacrifice*, EI = *Emotional Inhibition*, US = *Unrelenting Standards/Hypercriticalness*, ET = *Entitlement/Grandiosity*, ISC = *Insufficient Self-Control/Self-Discipline*, RS = *Approval-Seeking/Recognition-Seeking*, PE = *Negativity/Pessimism*, PU = *Punitiveness*.
